# The effect of tranexamic acid on the reduction of intraoperative and postoperative blood loss and thromboembolic risk in patients with hip fracture

**DOI:** 10.1515/med-2022-0482

**Published:** 2022-04-29

**Authors:** Ivan B. Stojadinovic, Branko M. Ristic, Dragan R. Knezevic, Zoran S. Milenkovic, Nikola S. Prodanovic, Nenad R. Zornic, Jelena B. Milosevic

**Affiliations:** Department of Spinal Surgery, Clinic for Orthopedics and Traumatology, University Clinical Center Kragujevac, Kragujevac, 34000, Serbia; Department of Surgery, Faculty of Medical Sciences, University of Kragujevac, Svetozara Markovica St. 69, Kragujevac, 34000, Serbia; Vascular Surgery Department, University Clinical Center Kragujevac, Kragujevac, 34000, Serbia; Department of Anesthesia and Intensive Care, University Clinical Center Kragujevac, Kragujevac, 34000, Serbia; Department of Physical Medicine and Rehabilitation, Faculty of Medical Sciences, University of Kragujevac, Kragujevac, 34000, Serbia; Department of Traumatology, Clinic for Orthopedics and Traumatology, University Clinical Center Kragujevac, Kragujevac, 34000, Serbia; Department of Alloartoplastic Surgery, Clinic for Orthopedics and Traumatology, University Clinical Center Kragujevac, Kragujevac, 34000, Serbia

**Keywords:** tranexamic acid, hip fracture, blood loss, thromboembolism

## Abstract

The aim of this study is to determine whether the use of tranexamic acid (TXA) in patients with hip fracture reduces intraoperative and postoperative blood loss, and on the other hand, whether it increases thromboembolic risk. The study was performed on patients with hip fracture for a period of one year. Patients were divided into two groups (1:1): the first group receiving TXA and the second group receiving placebo. The amount of blood aspiration during the surgery was measured as well as drainage in the postoperative period of 24 h. The occurrence of deep vein thrombosis (DVT) was monitored before and after the surgery by ultrasound of the lower extremities. The amount of total blood loss was two times lower in patients who received TXA (291.8 ± 65.5 mL of blood vs 634.7 ± 150.5 mL of blood). Among the patients who developed DVT, one patient was from the group that did not receive TXA, and two patients were from the group that received TXA. The use of TXA in patients with hip fracture significantly reduces intraoperative and postoperative blood loss, without a significant thromboembolic risk.

## 1 Introduction

Fractures of the proximal part of the femur represent a major clinical problem due to numerous complications and high mortality. According to some authors, mortality in these patients in the first year is 25% [1,2,3]. One of the main problems in people with hip fracture is blood loss [1,2]. It occurs immediately after the injury, although it can also occur as a complication of surgical treatment. This often leads to postoperative anemia [4] and the need for blood transfusion. Transfusions of blood and blood derivatives carry with them the risk of transmitting various diseases, the occurrence of immune reactions, and the risk of developing postoperative infections [5]. In recent years, tranexamic acid (TXA) has been increasingly used as one way to reduce blood loss. Together with ε-aminocaproic acid and aprotinin, TXA belongs to antifibrinolytics [6]. On the other hand, there is a suspicion that the use of such preparations increases thromboembolic risk.

The aim of our study is to determine whether the use of TXA in patients with hip fracture affects the reduction of intraoperative and postoperative blood loss, hemoglobin and hematocrit values in patients after surgery, reduction of the need for blood transfusion, and whether it increases thromboembolic risk.

## 2 Materials and method

A prospective randomized clinical study lasting one year was conducted. The study included patients with hip fractures who were surgically treated at the Clinic for Orthopedics and Traumatology of the University Clinical Center Kragujevac. The study was approved by the ethics committee of our hospital. Written informed consent was obtained from each patient.

We divided the patients into two groups: the first group receiving TXA and the second group receiving placebo. TXA was administered in two equal doses. The first dose of 15 mg/kg body weight was given immediately before the surgery, and the second, in the same dose 3 h afterward. Randomization was carried out by the method of closed envelopes with the group number, which were opened immediately before the surgery.

### 2.1 Inclusion and exclusion criteria

The study included patients of both genders, over 18 years of age, with a confirmed diagnosis of hip fracture acquired in the last 24 h (fractures of the proximal part of the femur). Excluding factors are patients with polytrauma, impaired coagulation status, suspected hypersensitivity to TXA, patients with open fractures, long-term uninterrupted anticoagulant therapy, patients with a history of arterial or venous thrombosis or thromboembolic risk, patients with pathological fractures, patients who have suffered from deep vein thrombosis (DVT), pulmonary embolism (PE), cerebrovascular stroke or myocardial infarction in the last year, patients with disseminated intravascular coagulation, patients who have experienced subarachnoid hemorrhage, renal failure, severe liver diseases, pregnant, breastfeeding, or women taking oral contraceptives.

### 2.2 Outcome measurements

The following variables were recorded and analyzed for each patient: gender, age, type of fracture (the femur neck, trochanteric region), preoperative values of hemoglobin, hematocrit, D-dimer, blood pressure, the amount of blood aspiration during surgery, postoperative values of hemoglobin and hematocrit, number of blood units required for transfusion in the postoperative period of 14 days, total blood loss (measured by Gross equation [7]), presence of DVT diagnosed by ultrasound of the lower extremities, and diagnosed lung embolization (if in doubt, we performed lung scintigraphy and multi-slice computed tomography diagnostics).

The indication for postoperative transfusion was a hemoglobin value below 80 g/L. In the preoperative and postoperative period, an ultrasound examination of blood vessels of the lower extremities was performed in all patients to possibly detect asymptomatic DVT. Surgical treatment involved performing surgeries by the same surgical team that represents standard procedures for a certain type of fracture (total hip endoprosthesis, partial hip endoprosthesis, proximal femoral nail, and dynamic hip screw [DHS]). Patients follow-up was three months after the surgery.

### 2.3 Statistical analysis

Statistical data processing was performed using SPSS 20 statistical software. In the data analysis, the chi-square test (*χ*²) or the Fisher test with a low frequency of certain categories was used. The significance of the difference in the values of the continuous variables between cases and controls was tested by the student’s *T* test for the independent samples (in the case of normal distribution) or the Mann–Whitney *U* test (in the case of the absence of normal distribution).

## 3 Results

The study included 80 patients, 34 (42.5%) male and 46 (57.5%) female, and the average age of 75.4 ± 82 years. A fracture of the femur neck was detected in 41 patients (51.3%), while the extracapsular fracture of the upper part of the femur was found in 39 patients (48.7%). The most commonly used surgical technique for hip fracture treatment was osteosynthesis of the proximal part of the femur with the proximal femoral nail antirotation (PFNA) technique, in every third patient, and then partial hip arthroplasty with the implantation of a biarticular endoprosthesis, in every fourth patient. Hip arthroplasty with the implantation of a unipolar endoprosthesis (Austin Moore) was the least commonly used, in every tenth patient.

Forty patients, 23 (57.5%) male and 17 (42.5%) female, the average age of 74.2 ± 86 years, received TXA. The use of TXA is similar in both types of the femur fractures (*χ*² = 1.25, df = 1, *p* > 0.05). Among the operated patients who received TXA, 55% (*n* = 22) had a trochanteric femur fracture, and 45% had a femoral neck fracture (*n* = 18). In patients not receiving TXA, 57.5% (*n* = 23) had a femoral neck fracture, and 42.5% (*n* = 17) had a fracture of the proximal part of the femur.

The values of the basic laboratory parameters before surgery are mostly within the reference limits in both groups of patients that were examined. The exception is the value of hemoglobin and systolic blood pressure. The mean value of preoperative hemoglobin in patients who received TXA was 114.5 ± 15.2 g/L, and in patients who did not 121.9 ± 11.3 g/L. The mean value of preoperative systolic blood pressure in patients given TXA was lower by 4 mmHg (121.25 ± 9.4 mmHg vs 125.4 ± 7 mmHg) (Table 1).

**Table 1:** Preoperative and postoperative values of the basic laboratory parameters in relation to the use of TXA

		Preoperative values					Postoperative values				
Parameters		TXA		TXA			TXA		TXA		
		Yes		No			Yes		No		
	*n*	*x*	sd	*x*	sd	*p*	*x*	sd	*x*	sd	*p*
Erythrocytes	40	3.83	0.52	3.79	0.56	>0.05	3.48	0.56	2.76	0.53	<**0.01**
Platelets	40	288.33	80.78	256.38	63.14	>0.05	287.3	79.75	227.78	64.1	<**0.01**
Hemoglobin	40	114.5	15.24	121.85	11.26	>0.05	108.45	15.32	87.45	11.21	<**0.01**
Hematocrit	40	0.33	0.04	0.33	0.047	>0.05	0.327	0.04	0.233	0.07	<**0.01**
Prothrombin time	40	1.08	0.08	1.09	0.15	>0.05	1.09	0.08	1.07	0.08	<0.05
aPTT	40	26.13	3.52	26.67	3.46	>0.05	26.64	4.79	27.19	4.39	<0.05
Fibrinogen	40	6.63	1.96	6.57	1.82	>0.05	6.89	2.15	7.82	2.37	<0.05
Systolic blood pressure	40	121.25	9.39	125.4	7.02	>0.05	116.8	8.1	107.5	8.2	**<0.01**
Diastolic blood pressure	40	75.75	7.47	77.88	6.19	>0.05	72	6.3	65.1	5.1	**<0.01**

**Figure 1:**
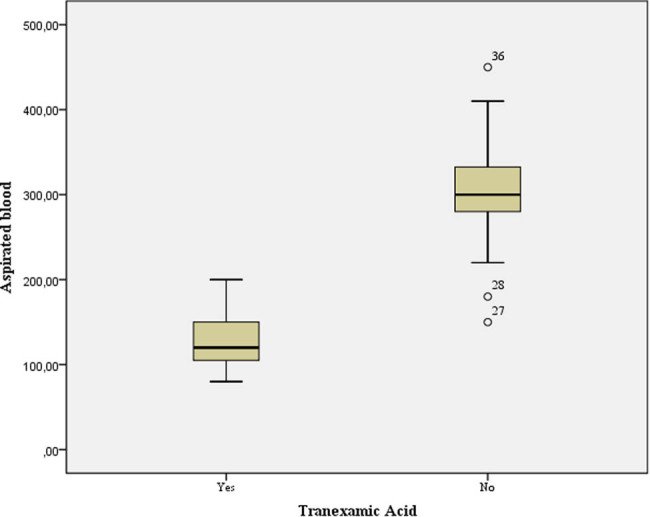
Blood aspiration in the analyzed groups.

The mean amount of blood aspiration in patients receiving TXA was 133 ± 32.1 mL vs 305.8 ± 60.3 mL in patients receiving placebo (Figure 1) (Table 2).

**Table 2:** Intraoperative blood loss by type of fracture and application of tranexamic acid (TXA)

Type of fracture	Tranexamic acid	*n*	*x̄*	sd	*p*
Fractura colli femoris	Yes	18	138.3	29.8	<**0.01**
	No	23	318.9	51.6	
Fractura trohanterica femoris	Yes	22	128.6	33.8	<**0.01**
	No	17	287.9	67.8	

**Figure 2:**
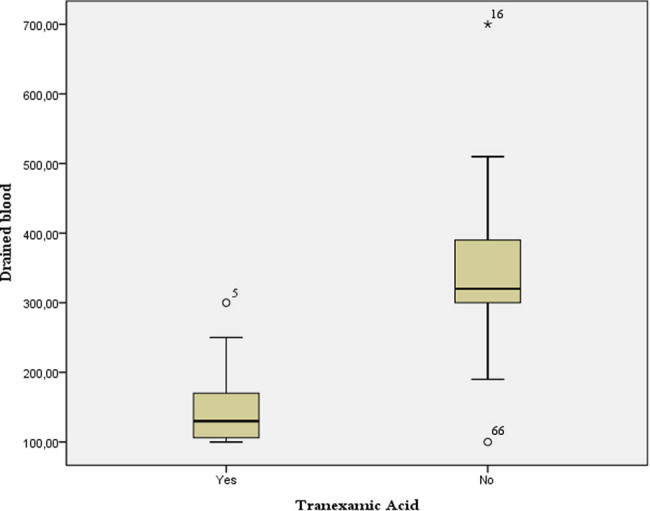
Drainage in the analyzed groups.

On average, 248.2 ± 127.9 mL of blood was drained after the surgery. Patients who had received TXA statistically lost less blood in the postoperative period compared with patients who had not received TXA (*t* = −11.2, df = 58.6, *p* < 0.01). The mean amount of drainage in patients who received TXA was 147.2 ± 51.5 mL compared with 346.8 ± 100.1 mL in patients who did not receive TXA (Figure 2).

**Figure 3:**
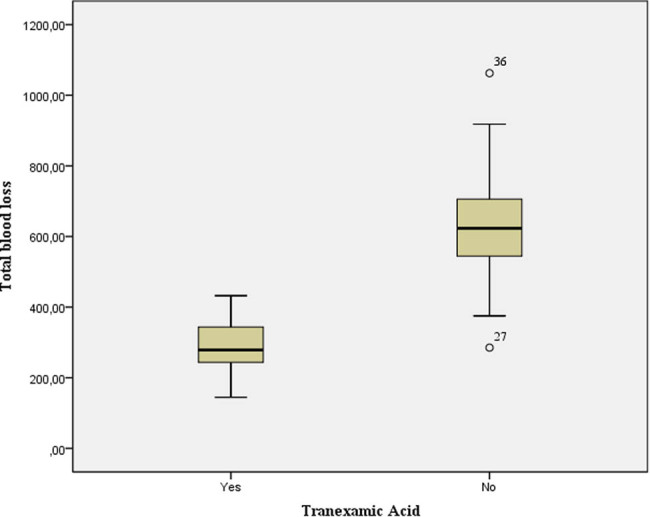
Total blood loss in the analyzed groups.

The average total blood loss, measured using the Gross equation, was 463.2 ± 207.5 mL of blood. The amount of total blood loss was two times lower in patients who received TXA (291.8 ± 65.5 mL of blood vs 634.7 ± 150.5 mL of blood), which is a statistically significant difference (*t* = −13.21, df = 53.2, *p* < 0.01) (Figure 3).

Comparison of the values of the basic laboratory parameters after surgery of the examined groups of patients indicates that the patients not receiving TXA experienced a statistically significant decrease in values of the following: erythrocyte from 3.48 ± 0.56 to 2.76 ± 0.53 (*t* = 5.84, df = 78, *p* < 0.01), platelets from 287.3 ± 79.75 to 227.78 ± 64.1 (*t* = 3.68, df = 78, *p* < 0.01), hemoglobin from 108.45 ± 15.32 to 87.48 ± 11.21 (*t* = 6.99, df = 71.4, *p* < 0.01), hematocrit from 0.327 ± 0.04 to 0.233 ± 0.07 (*t* = 12.26, df = 79, *p* < 0.01) and consequently systolic blood pressure from 116.8 ± 8.1 to 107.5 ± 8.2 (*t* = 5.1, df = 78, *p* < 0.01), and diastolic blood pressure from 72 ± 6.3 to 65.1 ± 5.1 (*t* = 5.36, df = 78, *p* < 0.01) (Table 1).

The need for blood transfusion was in 13.8% of patients (*n* = 11), two male and nine female, and the average age was 78.1 ± 7.9 years. Two patients from the first group (18.2%), who received TXA, needed a blood transfusion, while nine patients from the second group (81.8%), who did not received TXA, needed a blood transfusion.

Deep vein thrombosis (DVT) occurred in three patients (two men/one woman). One patient developed DVT after femoral neck surgery and two patients after trochanteric femoral region surgery. Of these three patients with DVT, two were treated with TXA and one was not.

## 4 Discussion

Many studies describe the use of TXA in orthopedic surgery, primarily in arthroplasty and spinal surgery. Some authors (Engel et al. [8]) have failed to demonstrate a significant reduction in postoperative blood loss and the need for blood transfusion in the study and control groups. In contrast, other authors have succeeded in demonstrating a significant difference [9]. Despite proven efficacy in elective orthopedic surgery, the optimal dosage and timing of TXA administration is still being debated. Moreover, the efficacy and side effect profile of TXA in patients with fractures remain unclear [10]. By examining the effect of TXA in patients with hip fractures, we obtained the significant results on the safety and efficacy of this drug in a population that, compared to that in elective surgery, is always with higher comorbidity and of greater age.

It is important to note that hip fractures are not all the same in terms of the pattern of injury or surgical treatment. Extracapsular hip fractures are associated with higher total blood loss than intracapsular hip fractures [11]. Therefore, the effect of TXA can vary with different hip fractures as well as with different surgical procedures. Extracapsular hip fractures are usually treated with an intramedullary nail or dynamic compression screw. In a randomized controlled trial of 72 patients, Tengberg et al. examined the effect of TXA in patients with extracapsular hip fractures who underwent operative fixation with a short intramedullary nail. Their protocol consisted of a preoperative dose of 1 g TXA followed by a 24 h postoperative infusion of 3 g TXA. TXA reduced total blood loss by 600 mL and it also reduced the risk of blood transfusion, without a significant increase in venous thromboembolic events, to 90 days, postoperatively. Similarly, some authors found that one preoperative dose of TXA reduced the total blood loss in the study of 60 patients with extracapsular hip fractures treated with dynamic compression screw [11].

Studies in patients who suffered a hip fracture exist, but the results obtained are much less convincing than in studies with patients who underwent total arthroplasty of the hip or knee, especially in relation to the occurrence of thromboembolic risk [12,13]. Patients with hip fractures are quite different from patients who had elective hip arthroplasty. They are generally older and at higher postoperative risk [13]. Recently, several papers showing a sufficient level of safety in the use of TXA in patients with hip fractures [10,14,15,16,17] have been published.

In our study, in addition to the effect of TXA on hip fractures in total, the effect of intracapsular and extracapsular fractures was examined separately. Intraoperative blood loss in intracapsular fractures was slightly higher (138.3 mL on average in patients who received and 318.9 mL on average in patients who did not receive TXA) than in extracapsular fractures (128.6 and 287.9 mL). It can be explained by various surgical techniques, because hip arthroplasty, which is mainly performed in intracapsular fractures, is certainly more invasive than the PFNA and DHS techniques. There are concerns about a hypothetical increase in the thromboembolic risk, DVT, and PE following the systemic administration of TXA in major orthopedic surgeries. There is a statistically significant difference in intraoperative blood loss depending on whether the patient has been treated with TXA, regardless of the type of fracture (Table 2). Massimo Francini et al. evaluated intravenous use of TXA in major orthopedic surgeries. They observed that the overall incidence of venous thromboembolism is 86 in 4,174 (2.1%) patients with intravenous infusion of TXA infusion and 55 in 2,779 (2.0%) patients in the control group [18].

Jinwei Xie and co-workers performed ultrasonography only if DVT was suspected, while computed tomography was performed for clinically suspected cases of PE. The overall erythrocyte transfusion rate was 16.75% (102 patients), with transfusion of at least 1 erythrocyte unit given to 25 patients in the TXA group (8.65%) and 77 in the control group (24.06%, *p* < 0,001). This corresponds to a significant relative risk reduction of 70% (odds ratio (OR) 0.299, 95% confidence interval (CI) 0.184–0.485). TXA was associated with a 17.15% reduction in total blood loss and a 64.05% reduction in erythrocyte transfusion rate in this study [19] Studies have shown trends toward a higher incidence of postoperative vascular events with TXA in patients with hip fracture (16% vs 6%, *p* = 0.10) [20] and higher 90-day mortality with TXA in patients with extracapsular fracture (27.2% vs 10.2%, *p* = 0.07) [21]. In our study, ultrasonography was performed in all patients, due to possible asymptomatic DVT. The results showed that there was no statistically significant difference in the occurrence of the thromboembolic risk in patients who received TXA and those who did not. Farrow and co-workers conducted a systematic review of the use of TXA in hip fracture surgery in 2016. The authors believed that there was the evidence of moderate quality showing that TXA could reduce blood loss in hip fracture surgery and there was the evidence of lower quality indicating that TXA did not increase the thromboembolic risk [22]. The results of our study certainly support the efficacy of TXA in hip fracture (extra and intracapsular), with good drug safety in relation to potential thromboembolic complications.

## 5 Conclusion

The use of TXA in patients with hip fracture (extracapsular and intracapsular) significantly reduces the amount of blood aspiration during surgery and after surgery. The total blood loss was 2.5 times lower in patients who received TXA compared with the control group. A decrease in the value of erythrocytes, hemoglobin, and hematocrit after hip fracture surgery, in both groups and with all surgical methods, was also detected. Patients who received TXA had a reduced need for blood transfusions. These patients also had a smaller postoperative decrease in the values of basic laboratory parameters compared with patients who did not receive TXA. Finally, the use of TXA is not associated with an increased risk of thromboembolism (thromboembolic events) in this type of patients.

## Abbreviations

TXA: tranexamic acid
MSCT: multi-slice computed tomography
PFNA: *(proximal femoral nail antirotation)* antirotation intramedullary nail for the proximal part of the femur
DHS: dynamic hip screw
BHP: *(biarticular hip prosthesis)* partial biarticular hip prosthesis
AM: *(Austin Moore)* Austin Moore partial hip prosthesis
VTE: venous thromboembolism
DVT: deep vein thrombosis
CVI: *cerebrovascular insult*

## References

[1] Prodovic T, Ristic B, Vucetic D, Ignjatovic-Ristic D. The impact of gender differences on mortality in elderly patients after hip fracture. Vojnosanit Pregl. 2018;75(9):918–25. 10.2298/VSP161122022P. https://aseestant.ceon.rs/index.php/vsp/article/view/12478/16419.

[2] Prodovic T, Ristic B, Rancic N, Bukumiric Z, Stepanovic Z, Ignjatovic-Ristic D. Factors influencing the six-month mortality rate in patients with a hip fracture. Zdr Varst. 2016;55(2):102–7. 10.1515/sjph-2016-0015. https://www.ncbi.nlm.nih.gov/pmc/articles/PMC4845770/pdf/sjph-2016-0015.pdf. PMC4845770 27284379

[3] Davidson CW, Merrilees MJ, Wilkinson TJ, McKie JS, Gilchrist NL. Hip fracture mortality and morbidity- can we do better? N Zealand Med J. 2001;114(1136):329–32. 11548098

[4] Foss NB, Kehlet H. Hidden blood loss after surgery for hip fracture. J Bone Jt Surg Br. 2006;88(8):1053–9. 10.1302/0301-620X.88B8.17534. 16877605

[5] Sadeghi M, Mehr-Aein A. Does a single bolus dose of tranexamic acid Reduce blood lose and transfusion requirements during hip fracture surgery? A prospective randomized double blind study in 67 patients. Acta Medica Iran. 2007;45(6):437–42.

[6] Eubanks JD. Antifibrinolytics in major orthopaedic surgery. J Am Acad Orthop Surg. 2010;18:132–8. 20190103

[7] Tian S, Li H, Liu M, Zhang Y, Peng A. Dynamic analysis of perioperative hidden blood loss in intertrochanteric fractures. Clincal Appl Thrombosis/Hemostasis. 2019;25:1–5. 10.1177/1076029618823279. https://journals.sagepub.com/doi/full/10.1177/1076029618823279. PMC6714944 30803260

[8] Engel JM, Hohaus T, Ruwoldt R, Menges T, Jurgensen I, Hemplemann G. Regional haemostatic status and blood requirements after total knee arthroplasty with and without tranexamic acid or aprotinin. Anesth Analg. 2001;92(3):775–80. 10.1097/00000539-200103000-00041 11226117

[9] Gandhi R, Evans HM, Mahomed SR, Mahomed NN. Tranexamic acid and the reduction of blood loss in total knee and hip arthroplasty: a meta-analysis. BMC Res Notes. 2013;6:184. 10.1186/1756-0500-6-184. https://www.ncbi.nlm.nih.gov/pmc/articles/PMC3655041/pdf/1756-0500-6-184.pdf. PMC3655041 23651507

[10] Gausden EB, Garner MR, Warner SJ, Levack A, Nellestein AM, Tedore T, et al. Tranexamic acid in hip fracture patients: a protocol for a randomised, placebo controlled trial on the efficacy of tranexamic acid in reducing blood loss in hip fracture patients. BMJ Open. 2016;6:e010676. 10.1136/bmjopen-2015-010676. https://www.ncbi.nlm.nih.gov/pmc/articles/PMC4916621/pdf/bmjopen-2015-010676.pdf. PMC4916621 27329438

[11] Cheung ZB, Anthony SG, Forsh DA, Podolnick J, Zubizarreta N, Galatz LM, et al. Utilization, effectiveness, and safety of tranexamic acid use in hip fracture surgery: A population-based study. J Orthop. 2020;20:167–72. 10.1016/j.jor.2020.01.040. https://www.ncbi.nlm.nih.gov/pmc/articles/PMC6997115/pdf/main.pdf. PMC6997115 32025142

[12] Krebs NM, VanWagner MJ, Marchewka T, Faraj U, Vitale CR. Tranexamic acid in the treatment of hip fractures: a clinical review. Spartan Med Res J. 2019;3(3):7026. 10.51894/001c.7026. https://www.ncbi.nlm.nih.gov/pmc/articles/PMC7746023/pdf/smrj_2019_3_3_7026.pdf. PMC7746023 33655149

[13] Ashkenazi I, Schermann H, Gold A, Lin R, Pardo I, Steinberg E, et al. Tranexamic acid in hip hemiarthroplasty. Injury. 2020;51(11):2658–62. 10.1016/j.injury.2020.07.061 32763019

[14] Tian S, Shen Z, Liu Y, Zhang Y, Peng A. The effect of tranexamic acid on hidden bleeding in older intertrochanteric fracture patients treated with PFNA. Injury. 2018;49(3):680–4. 10.1016/j.injury.2018.01.026. https://www.injuryjournal.com/article/S0020-1383(18)30026-3/fulltext. 29426608

[15] Baruah RK, Borah PJ, Haque R. Use of tranexamic acid in dynamic hip screw plate fixation for trochanteric fractures. J Orthop Surg (Hong Kong). 2016;24(3):379–82. 10.1177/1602400322. https://journals.sagepub.com/doi/pdf/10.1177/1602400322. 28031511

[16] Mohib Y, Rashid RH, Ali M, Zubairi AJ, Umer M. Does tranexamic acid reduce blood transfusion following surgery for inter-trochanteric fracture? A randomized control trial. J Pak Med Assoc. 2015;65(3):17–20. 26878513

[17] Lee C, Freeman R, Edmondson M, Rogers BA. The efficacy of tranexamic acid in hip hemiarthroplasty surgery: an observational cohort study. Injury. 2015;46(10):1978–82. 10.1016/j.injury.2015.06.039. 26190627

[18] Franchini M, Mengoli C, Marietta M, Marano G, Vaglio S, Pupella S, et al. Safety of intravenous tranexamic acid in patients undergoing majororthopaedic surgery: a meta-analysis of randomised controlled trials. Blood Transfus. 2018;16:36–43. 10.2450//2017.0219-17 PMC5770313 29337665

[19] Xie J, Hu Q, Huang Q, Chen G, Zhou Z, Pei F. Efficacy and safety of tranexamic acid in geriatric hip fracture with hemiarthroplasty: a retrospective cohort study. BMC Musculoskelet Disord. 2019;20(1):304. 10.1186/s12891-019-2670-5. https://www.ncbi.nlm.nih.gov/pmc/articles/PMC6598293/pdf/12891_2019_Article_2670.pdf. PMC6598293 31248398

[20] Zufferey PJ, Miquet M, Quenet S, Martin P, Adam P, Albaladejo P, et al. Tranexamic acid in hip-fracture surgery (THIF) study. Tranexamic acid in hip fracture surgery: a randomized controlled trial. Br J Anaesth. 2010 Jan;104(1):23–30. 10.1093/bja/aep314. PMID: 19926634. 19926634

[21] Baruah RK, Borah PJ, Haque R. Use of tranexamic acid in dynamic hip screw plate fixation for trochanteric fractures. J Orthop Surg (Hong Kong). 2016 Dec;24(3):379–82. 10.1177/1602400322, PMID: 28031511. 28031511

[22] Farrow LS, Smith TO, Ashcroft GP, Myint PK. A systematic review of tranexamic acid in hip fracture surgery. Br J Clin Pharmacol. 2016;82:1458–70. 10.1111/bcp.13079. PMC5099561 27492116

